# Ligament reconstruction in thumb carpometacarpal joint instability: A systematic review

**DOI:** 10.1016/j.jpra.2024.01.001

**Published:** 2024-01-12

**Authors:** I.C. Jongen, N.J. Nieuwdorp, C.A. Hundepool, F.S. Van Gelder, A.M. Schutter, J.M. Zuidam

**Affiliations:** Department of Plastic, Reconstructive and Hand Surgery, Erasmus MC, Dr. Molewaterplein 40, 3015 GE Rotterdam, the Netherlands

**Keywords:** Joint instability, Ligament reconstruction surgery, Tendon-looping procedures, Thumb carpometacarpal joint, Trapeziometacarpal joint

## Abstract

In thumb carpometacarpal (CMC) instability, laxity of the ligaments surrounding the joint leads to pain and weakness in grip and pinch strength, which predisposes the patient to developing CMC joint arthritis. Recent advancements in joint anatomy and kinematics have led to the development of various surgical reconstructive procedures. This systematic review outlines the available ligament reconstruction techniques and their efficacy in treating nontraumatic and nonarthritic CMC instability. Additionally, we aimed to provide evidence which specific ligament reconstruction technique demonstrates the best results. Four databases (Embase, MEDLINE, Web of Science, and Cochrane Central) were searched for studies that reported on surgical techniques and their clinical outcomes in patients with nontraumatic and nonarthritic CMC instability. Twelve studies were analyzed for qualitative review, including nine different surgical ligament reconstruction techniques involving two hundred and thirty thumbs. All but one of the reported techniques improved postoperative pain scores and showed substantial improvement in pinch and grip strength. Complication rates varied between 0% and 25%. The included studies showed that ligament reconstruction effectively alleviated the patients’ complaints regarding pain and instability, resulting in overall high patient satisfaction. Nevertheless, drawing definitive conclusions regarding the superiority of any ligament reconstruction technique remains challenging owing to the limited availability of homogeneous data in the current literature.

## Introduction

The trapezium bone and base of the first metacarpal together form the double saddle-shaped thumb carpometacarpal (CMC) joint. The geometry of the thumb CMC joint allows for multiplanar motions but offers relatively little osseous support. Therefore, joint stability mainly depends on its surrounding capsuloligamentous structures.^1^ In CMC joint instability, laxity of these stabilizing ligaments leads to incongruity of the CMC joint, which results in increased shear forces on the joint and subsequent synovitis. Patients typically present with pain in the thenar eminence and weakness in grip and pinch strength. CMC joint instability is often observed as a manifestation of generalized joint hypermobility. The laxity of ligaments may also result from trauma, trapezial dysplasia, or metabolic disorders such as Ehlers-Danlos syndrome.^2^, ^3^, ^4^ If left unaddressed, the ongoing processes of joint incongruity and synovitis can cause the dorsal translation of the first metacarpal, further increasing the focal contact pressure of the joint, and ultimately predisposing the patient to developing CMC joint arthritis.^5^, ^6^, ^7^, ^8^, ^9^, ^10^ Surgical intervention is indicated when conservative treatment fails to slow the progression of instability or if symptoms persist.^3^^,^^4^

The stabilizing ligaments of the CMC joint have been extensively studied in terms of their anatomy and relative contribution to joint stability.^1^^,^^5^^,^^11^, ^12^, ^13^ Over the past decades, several surgical techniques have been proposed to improve joint stability and kinematics with the aim of reconstructing or strengthening the affected capsuloligamentous structures.^14^, ^15^, ^16^, ^17^, ^18^ Early ligament reconstruction techniques primarily focused on reconstructing the joint's anterior oblique ligament (AOL), as it was considered the primary stabilizer.^9^^,^^10^^,^^13^^,^^19^ In 1973, the “volar ligament reconstruction” technique was introduced by Eaton-Littler, in which a strip of the flexor carpi radialis (FCR) tendon was passed through an extra-articular drill hole at the base of the first metacarpal, looped around the remaining FCR, and secured over the radial side of the joint.^6^ Eaton and Littler's ligament reconstruction remains the most studied and used procedure to this day. Nevertheless, controversy exists as to which specific ligament contributes the most to CMC joint stability and recent anatomical and biomechanical studies have emphasized the importance of the dorsoradial ligament (DRL) in CMC joint stability.^3^^,^^5^^,^^11^^,^^12^^,^^20^, ^21^, ^22^, ^23^, ^24^, ^25^, ^26^ Since then, various reconstruction and augmentation techniques focusing on the dorsal aspect of the CMC joint have been developed.^27^^,^^28^ Although the debate on the relative importance of the ligaments persists, the AOL, DRL, and intermetacarpal ligament are generally considered the prime stabilizers of the CMC joint.

Despite these recent advances in anatomical knowledge and surgical approaches, current literature fails to provide a consensus on the optimal ligament reconstruction technique. Hence, we aimed to review the available literature for the surgical outcomes of various ligament reconstruction techniques used in nontraumatic and nonarthritic CMC instability. Additionally, we aimed to provide evidence supporting the superiority of a specific ligament reconstruction technique.

## Methods

A systematic review of the current literature on ligament reconstruction techniques in thumb CMC instability was conducted. Embase, MEDLINE, Web of Science, and Cochrane Central were searched on April 12, 2023 (see S1 for the search strategy). The systematic search was conducted in consultation with a medical information specialist and was performed in accordance with the Preferred Reporting Items for Systematic Reviews and Meta-analyses statement (Table S2).^29^

### Study selection

Two authors (I.C.J. and N.J.N.) independently selected studies that met the eligibility criteria based on the title and abstract. All studies were screened for the following inclusion criteria: clinical studies reporting the outcomes of ligament reconstruction techniques in thumb CMC instability, providing postoperative outcomes, and describing the techniques used. Studies that reported outcomes in patients with chondropathy of the CMC joint, metabolic or connective tissue diseases with gross instability (e.g., Ehlers-Danlos syndrome), congenital abnormalities, or trauma (e.g., Bennet's fracture) were excluded. Reviews, case reports, case series with less than five patients, descriptive studies, cadaver or animal studies, conference abstracts, poster presentations, and non-English and non-full text articles were also excluded. Furthermore, studies on thumb metacarpal extension osteotomy were excluded as these studies focused on the redistribution of the contact areas by altering the mechanical loading rather than achieving stability via ligament reconstruction.^30^ Disagreements between authors were resolved in consensus meetings.

### Data extraction and quality scoring

Data were extracted from the selected studies using a standardized data collection form. The collected variables included year of publication, study type, number of patients, surgical technique, reported outcomes measured, and time to follow-up. The primary outcome was patient-reported pain. Secondary outcomes included grip and pinch strength, patient satisfaction, Quick DASH scores, and complications. Quality assessment was performed using the study quality assessment tools of the National Institutes of Health (NIH). The NIH tool was used for assessing controlled intervention studies, observational cohort and cross-sectional studies, and before-and-after (pre-post) studies with no control group (Table S3). Postoperative complications were scored according to the International Consortium for Health Outcomes Measurement Complications in Hand and Wrist (ICHAW) conditions tool (Table S4).^31^ The strength of evidence of each study was assessed using the classification of strength of evidence by Jovell and Navarro-Rubio (Table 1; see S5 for the classification).^32^

### Statistical analysis

Mean postoperative visual analog scale (VAS) scores for pain were visualized using a forest plot. When not reported, standard deviations and confidence intervals were computed.^33^ Individual study weights were calculated based on the 95 percent confidence intervals. The overall effect was evaluated using the random effect model.

## Results

**Figure 1:**
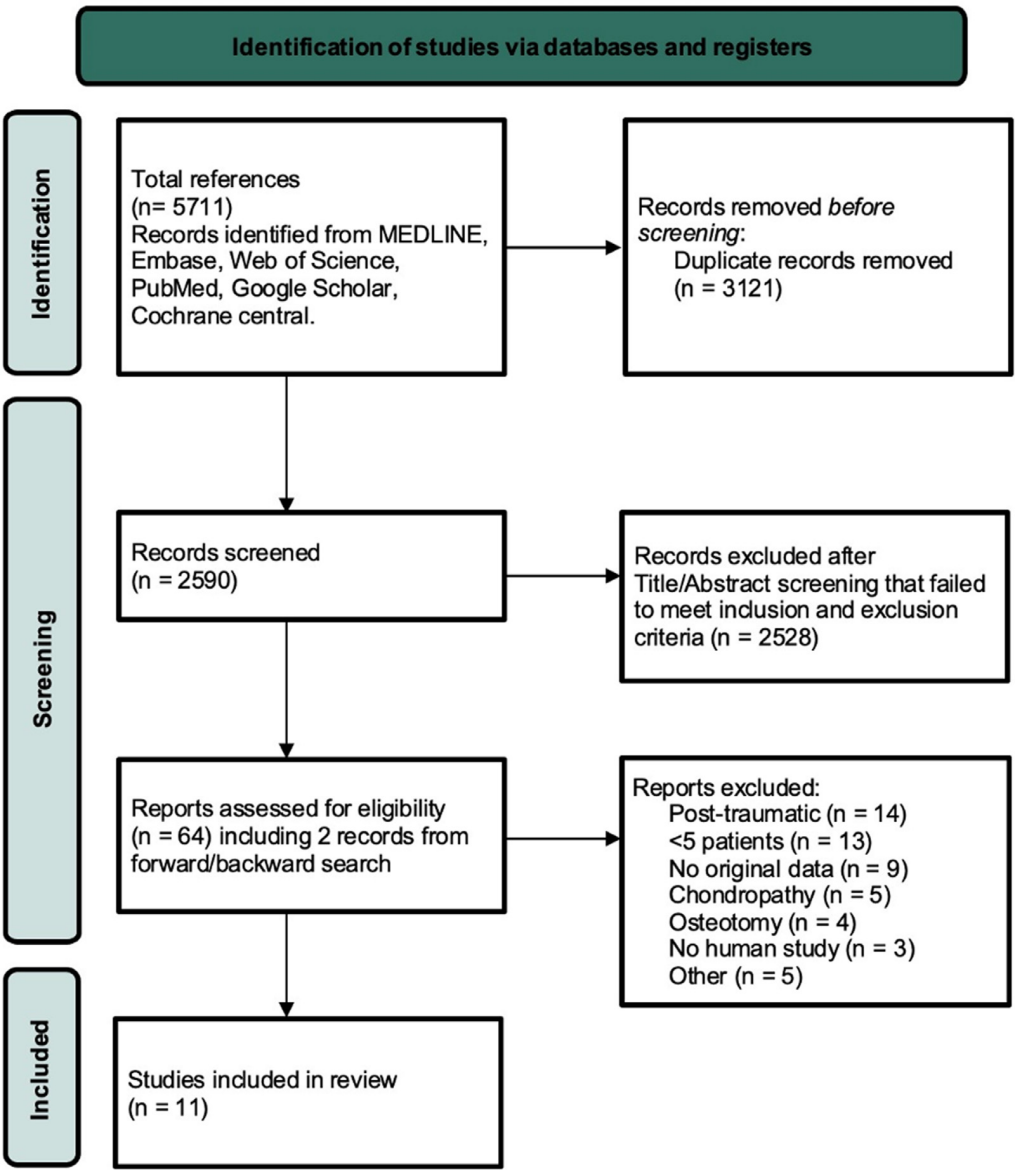
Flowchart representing the selection of included publications according to the preferred Reporting Items for Systematic Reviews and Meta-Analyses standards. *From:* Page MJ, McKenzie JE, Bossuyt PM, Boutron I, Hoffmann TC, Mulrow CD, et al. The PRISMA 2020 statement: an updated guideline for reporting systematic reviews. BMJ 2021;372:n71. doi:10.1136/bmj.n71.

The literature search yielded 5711 publications (Figure 1). After title and abstract screening and removal of duplicates, full texts of 62 publications were screened. Two additional publications were identified by hand-searching the reference lists.^34^^,^^35^ Eleven publications consisting of 12 studies that met the inclusion and exclusion criteria were analyzed for qualitative review, including one randomized controlled trial (RCT),^36^ six before-after studies,^34^^,^^36^, ^37^, ^38^, ^39^, ^40^ and five observational studies.^7^^,^^35^^,^^41^, ^42^, ^43^ These articles reported a total of 216 patients, 230 thumbs, and 9 different surgical techniques (Tables 1 and 2). The included studies reported the surgical outcomes with long-term follow-up ranging from 12 months to 15 years.

**Table 1:** Study and patient characteristics of all included studies by tendon or technique used.

Studies per surgical technique	LoE	No. of thumbs (No. of patients)	Female (%)	Age Mean (SD)	Follow-up (Years) Mean (range or SD)
**TENDON LOOPING**					

Eaton and Littler					
Eaton, 1984*	VI	8 (8)	NR	NR	7 (4–13)
Freedman, 2000	VI	24 (19)	68.4	9 (18)	15 (10–23)
Lane, 2001	VI	37 (35)	82.8	33	5.2 (1–17)
Spekreijse, 2016-RCT	III	8 (8)	100	35.9 (13)	1
Spekreijse 2016-Cohort	VI	57 (54)	94.4	34 (13)	1
ECRL tendon					
Biddulph, 1985	VI	10 (10)	50	18	3.5 (1–7)
Spekreijse, 2016 - RCT	III	8 (8)	87.5	35.9 (5)	1
APL tendon slip					
Stauffer, 2020	VI	15 (12)	91.7	23.2 (9.3)	3.5 (1.3 – 5.8)
FCR tendon					
Pecache, 2022	VI	16 (15)	NR	48 (10)	≥ 1⁎⁎
PL tendon and TCL flap					
Kato, 2022	VI	6 (6)	83.3	44 (17 – 75)	1.7 (1.5–1.9)

**CAPSULOPLASTY**					

Dorsoradial capsulodesis					
Koehler, 2023	VI	13 (13)	84.6	39 (10)	3.6 (0.8–12.3)
Dorsal stabilization and internal brace					
Kronlage, 2023	VI	11 (11)	90.9	34 (10)	2.2 (0.88)
Thermal Capsulorrhaphy					
Chu, 2009	VI	7 (7)⁎⁎⁎	42.9	43.6 (13.5)	3.7 (1.6)

⁎ Only patients with Eaton stage 1 were included in this study.

⁎⁎ Minimum follow-up of 1 year.

⁎⁎⁎ Only patients with nontraumatic instability were included in this study.

**Table 2:** Description of the included surgical techniques.

Article	Technique	
Eaton, 1984	Eaton and Littler	Distally attached FCR tendon slip is routed through a bone tunnel at the base of the first metacarpal under the insertion of the APL, looped around the remaining FCR, and secured over the radial side of the joint.
Freedman, 2000		
Lane, 2001		
Spekreijse, 2016		

Biddulph, 1985	ECRL	Tendon slip insertion is left intact. The ECRL slip is then looped around the first metacarpal across the palmar surface, under the APL and EPB and adductor pollicis, and then sutured to itself.

Chu, 2009	Thermal capsulorrhaphy	Arthroscopic procedure using a radiofrequency shrinkage probe, which is swept over the volar ligaments.

Spekreijse, 2016	ECRL	Distally attached ECRL tendon slip is passed through a bone tunnel at the base of the first metacarpal and back through a tunnel in the trapezium bone and reattached to itself at the base of the second metacarpal.

Stauffer, 2020	APL	Tendon slip insertion is left intact and routed through two deep transverse tunnels under the dorsal ligaments and joint capsule in a figure-of-eight fashion.

Koehler, 2023	Dorsoradial capsulodesis	DRL and joint capsule are incised. The reduced joint and the redundant ligament and capsule are overlapped and imbricated.

Pecache, 2022	FCR	A nonabsorbable suture is weaved through the distally attached FCR tendon slip, which is then routed through a bone tunnel in the first metacarpal, sutured to the APL insertion, routed through a second parallel bone tunnel in the trapezium in a figure-of-eight fashion, and sutured to itself.

Kato, 2022	PL and TCL	The rectangular radially based TCL flap is anchored to the insertion of the AOL at the metacarpal base. PL graft is attached to the trapezium, looped around and sutured to the APL and ECRL, and then sutured to the reconstructed DRL.

Kronlage, 2023	Dorsal stabilization with internal brace	Imbrication of the dorsal capsule followed by suture tape placement over the joint using suture anchors.

### Surgical techniques

The included studies described surgical techniques that aimed to achieve stability of the thumb CMC joint either by reconstructing the affected ligaments using a tendon strip^7^^,^^35^^,^^36^^,^^38^, ^39^, ^40^, ^41^^,^^43^ or by reinforcing the joint capsule (Table 2).^34^^,^^37^^,^^42^ In the included studies, the Eaton and Littler technique was the most studied procedure.^7^^,^^26^^,^^38^^,^^39^ Pecache and Tsai described a tendon looping technique similar to the volar ligament reconstruction, but the FCR strip was routed through a second bone tunnel in the trapezium and secured in a figure-of-eight fashion.^35^ The publication by Spekreijse et al. reported two studies: a cohort study on the Eaton and Littler technique and an RCT comparing the volar Eaton and Littler to a dorsal approach using the extensor carpi radialis longus (ECRL) tendon in a similar fashion.^36^ Biddulph also used the ERCL tendon; however, without using bone tunnels.^41^ Kato and Nomura reconstructed multiple ligaments using the palmaris longus (PL) dorsally and the transverse carpal ligament volarly.^40^ Stauffer et al. enhanced the dorsal ligaments and joint capsule by creating two deep transverse tunnels through the dorsal joint capsule through which the APL tendon was routed.^43^ In the studies focusing on joint capsule enhancement, Chu et al. performed a thermal capsulorrhaphy of the volar ligaments and joint capsule using a radiofrequency shrinkage probe, whereas Kronlage et al. and Koehler and Rayan imbricated the dorsal joint capsule.^34^^,^^37^^,^^42^ Additionally, Kronlage et al. employed an internal brace anchored to the trapezium and the first metacarpal base.^34^

### Outcome measurements

#### Pain

Eight studies incorporated the VAS scores as part of their outcome measurements, and five studies reported the pre- and postoperative VAS scores (Table 3).^7^^,^^34^^,^^37^^,^^36^^,^^40^^,^^42^^,^^43^ The tendon-looping procedure using the PL tendon and TCL flap reported the most notable improvement in mean VAS scores, which improved from 6 (range 5–7) preoperatively to 0.5 (range 0–1) postoperatively.^40^ Dorsal stabilization using an internal brace,^34^ volar thermal capsulorrhaphy,^37^ and the Eaton and Littler technique by Spekreijse et al.^36^ demonstrated significant decrease in postoperative VAS scores. However, in the RCT by Spekreijse et al., the dorsal approach using the ECRL exhibited a significant increase in VAS scores 3 months postoperatively, which resulted in the premature termination of the RCT.^36^ Contrarily, the other study that used the ECRL tendon in a dorsal approach without bone tunnels reported postoperative pain relief in 80% of the patients.^41^

**Table 3:** Pre- and postoperative visual analog scores for pain per technique.

Studies per surgical technique	No. of thumbs (No. of patients)	Pre-op VAS (0-10) Mean (SD or range)	Post-op VAS (0-10) Mean (SD or range)
**TENDON LOOPING**			

Eaton and Littler			
Freedman, 2000	24 (19)	NR	3.1 (1–6.5)
Spekreijse, 2016–RCT	8 (8)	5.85 (1.1)*	2.55 (0.55)*
Spekreijse, 2016–Cohort	57 (54)	6.13 (0.31)*	2.35 (0.59)*
ECRL tendon			
Spekreijse, 2016–RCT	8 (8)	3.03 (0.29)*	4.3 (0.79)*
APL tendon			
Stauffer, 2020	15 (12)	NR	1.1 (2.2)
PL tendon and TCL flap			
Kato, 2022	6 (6)	6 (5–7)	0.5 (0–1)

**CAPSULOPLASTY**			

Dorsoradial capsulodesis			
Koehler, 2023	13 (13)	NR	0.5 (0.9)
Dorsal stabilization and internal brace			
Kronlage, 2023	11 (11)	6.4 (1.8)	1.5 (1.8)
Thermal Capsulorrhaphy			
Chu, 2009⁎⁎	7 (7)	4.9 (0.7)	0.1 (0–1)

⁎ Originally VAS 0-100.

⁎⁎ Only patients with nontraumatic instability were included in this study.

**Figure 2:**
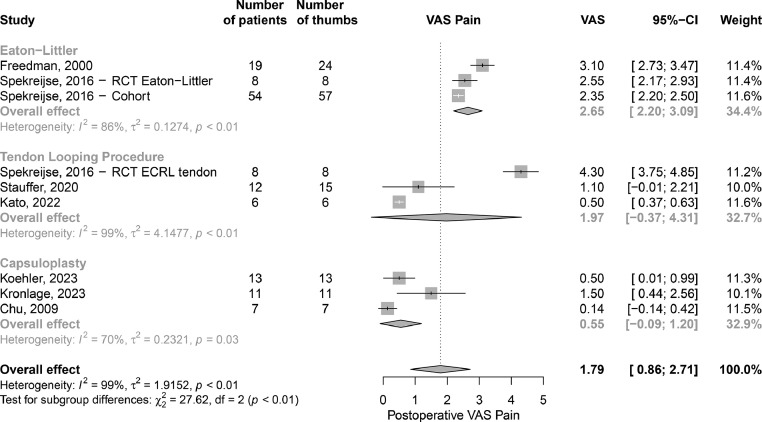
Postoperative visual analog scale scores for the long-term outcomes on pain (0 to 10 scale) per surgical technique.

The Eaton and Littler technique showed a pooled postoperative mean VAS score of 2.65 in 89 thumbs with a mean follow-up period ranging from 3 months to 15 years. The overall pooled mean postoperative VAS score for all the ligament reconstruction techniques was 1.97 with a mean follow-up period ranging from 3 months to 15 years, indicating a good clinical outcome (Figure 2). The remainder of the studies reported the percentage of patients (29% to 88%) who were pain-free postoperatively, with both studies using the Eaton and Littler technique.^7^^,^^38^

#### Pinch, key pinch, and grip strength

Pinch strength was assessed in six studies,^7^^,^^36^^,^^40^^,^^42^^,^^43^ key pinch in three,^36^^,^^40^ and grip strength in four (Table 4).^36^^,^^40^^,^^43^ Pinch and grip strength outcomes showed improvement for all techniques, with a statistically significant improvement in strength with the PL tendon and TCL flap technique, volar capsular thermorraphy, and the Eaton and Littler cohort by Spekreijse et al.^36^^,^^37^^,^^40^ In studies that did not report the strength in kilograms, the mean postoperative pinch strength was considered equal to or greater than the contralateral side in 100% of patients in one ECRL tendon looping study and two Eaton and Littler reconstruction studies.^7^^,^^38^ Remarkably, in the Eaton and Littler study by Lane and Henley, this was the case for only 41% of patients.^39^

**Table 4:** (Key) Pinch and grip strength.

Studies per surgical technique	No. of thumbs	Preoperative (kg) Mean (SD)	Postoperative (kg) Mean (SD)
**PINCH STRENGTH**			

Eaton and Littler			
Freedman, 2000	24	NR	6.5 (3–9.6)
Spekreijse, 2016–RCT	8	2.7 (0.5)	3.8 (0.3)
Spekreijse, 2016–Cohort	57	2.8 (0.2)	3.8 (0.3)
PL tendon and TCL flap			
Kato, 2022	6	2.5	4.1
APL tendon			
Stauffer, 2020	15	NR	5.6 (1.3)
Dorsoradial capsulodesis			
Koehler, 2023	13	NR	6.31 (1.50)

**KEY PINCH STRENGTH**			

Eaton and Littler			
Spekreijse, 2016–RCT	8	4.0 (0.4)	5.2 (0.6)
Spekreijse, 2016–Cohort	57	4.5 (0.3)	6.5 (0.4)
PL tendon and TCL flap			
Kato, 2022	6	4.2	6.1
ECRL tendon			
Spekreijse, 2016–RCT	8	4.6 (0.4)	5.2 (0.6)

**GRIP STRENGTH**			

Eaton and Littler			
Spekreijse, 2016–RCT	8	18.3 (3.4)	29.4 (1.8)
Spekreijse, 2016–Cohort	57	22 (1.4)	29 (0.4)
ECRL tendon			
Spekreijse, 2016–RCT	8	17 (1.6)	22.1 (3)
PL tendon and TCL flap			
Kato, 2022	6	20	26
APL tendon			
Stauffer, 2020	15	NR	24.6 (5.5)

#### Quick DASH and patient satisfaction

Three studies reported postoperative Quick DASH scores, ranging from 5.7 (SD 5.0) for the dorsal capsulodesis technique to a maximum of 30.79 (SD 23.21) for the FCR tendon-looping procedure.^35^^,^^42^ A significant improvement was observed for the dorsal stabilization using the internal brace technique.^34^ Postoperative DASH scores were reported in three studies and ranged from 13.3 (SD 11.3) for the APL tendon-looping procedure to 45.9 (SD 7.8) for the Eaton and Littler technique by Spekreijse et al.^36^^,^^43^ Seven studies reported patient satisfaction,^7^^,^^34^, ^35^, ^36^^,^^42^ ranging from 86.67% for the FCR tendon-looping procedure by Pecache and Tsai, to four studies reporting 100% patient satisfaction.^7^^,^^34^^,^^42^^,^^43^

#### Complications

The Eaton and Littler procedure, volar thermal capsulorrhaphy, and dorsal stabilization using an internal brace reported 0% complication rate (Table 5).^34^^,^^37^^,^^38^ The highest complication rate (25%) was observed in the RCT conducted by Spekreijse et al., followed by 19.3% in their Eaton and Littler cohort study.^36^ Five major complications (ICHAW grade 3) were reported for the Eaton and Littler technique.^7^^,^^36^^,^^39^ Overall, the most frequently reported complications included nerve irritation, nerve adherence, neuroma, sensory disturbances, tendon adhesions, and tendinitis.

**Table 5:** Complications reported in the included studies scored using the ICHAW classification.

Studies per surgical technique	Complication rate (%)	Type of complication	ICHAW classification
**TENDON LOOPING**			

**Eaton and Littler**			
Eaton, 1984*	0	NA	NA
Freedman, 2000		2 nerve irritation, 1 permanent pain from nerve adherence requiring surgical intervention	2 Grade 1, 1 Grade 3C
Lane, 2001	8.1	1 radial sensory nerve neuroma requiring transposition, 1 FCR adhesion requiring tenolysis, 1 RSD flare up	1 Grade 3, 2 grade 3C
Spekreijse, 2016–RCT	25	1 scar tenderness, 1 extensive infection requiring revision surgery and SSG	1 Grade 1, 1 Grade 3C
Spekreijse 2016 – Cohort	19.3	4 scar tenderness, 5 sensory disturbances, 1 tendinitis, 1 infection	9 Grade 1, 2 Grade 2
**ECRL tendon**			
Biddulph, 1985	NR	NR	NR
Spekreijse, 2016–RCT	25	1 ECRL tendinitis, 1 Quervain's disease	2 Grade 2
**APL tendon slip**			
Stauffer, 2020	6.7	1 recurrence of instability revision surgery not required	1 Grade 1
**FCR tendon**			
Pecache, 2022	9	1 stitch abscess, 1 irritation of the dorsal sensory nerve, 1 progress of CMC arthritis+	2 Grade 1 1 Grade 2
**PL tendon and TCL flap**			
Kato, 2022	NR	NR	NR

**CAPSULOPLASTY**			

**Dorsoradial capsulodesis**			
Koehler, 2023	7.7	1 cellulitis	1 Grade 2
**Dorsal stabilization and internal brace**			
Kronlage, 2023	0	NA	NA
**Thermal Capsulorrhaphy**			
Chu, 2009⁎⁎	o	NA	NA

⁎ Only patients with Eaton stage 1 were included in this study.

⁎⁎ Only patients with nontraumatic instability were included in this study.

+ Complication reported from operation for traumatic and nontraumatic instability (33 operations).

## Discussion

This study aimed to review the current literature on ligament reconstruction techniques in the surgical treatment of nontraumatic and nonarthritic thumb CMC instability. We aimed to provide a comprehensive overview of the available techniques and to evaluate their outcomes. The results from the included studies demonstrated that thumb CMC ligament reconstruction effectively addresses pain and instability, resulting in an overall high patient satisfaction. However, the limited availability of high-quality studies on CMC joint ligament reconstruction, characterized by limited sample sizes and the use of non-standardized outcome measures, made the comparison between individual techniques challenging.

The included studies clearly illustrated the shift of focus from reconstructing the volar ligaments to the dorsal ligaments. Except for the dorsal ECRL technique used by Spekreijse et al., favorable postoperative outcomes regarding pain and function were generally observed.^36^ Interestingly, Spekreijse et al. advised keeping the DRL intact during surgery, as they believed that damage to the richly innervated DRL complex might have contributed to the increase in postoperative pain.^36^ In contrast, the included studies that reported incision of the dorsal capsule^34^^,^^42^ or used the ECRL tendon as a graft^41^ demonstrated satisfactory postoperative results.

Despite the overall positive trend, more major complications (ICHAW grade 3) were reported for the Eaton and Littler technique.^7^^,^^36^^,^^39^ Concerns have been raised regarding its capacity to reconstruct the AOL given the differences in origin and force vector compared to the original ligament.^40^ Using bone tunnels might risk trapezium fracture and limit possible salvage options in cases requiring arthroplasty or arthrodesis whereas avoiding them might compromise secure tendon attachment.^35^^,^^40^^,^^43^ Other arguments made against tendon-looping procedures include increased exposure time, extensive open dissection, risk of iatrogenic injury to the sensory radial nerve, and potential weakening and rupturing of the donor tendon.^34^^,^^40^^,^^41^^,^^43^ However, the use of the FCR tendon-looping procedure demonstrated no adverse effects on the overall wrist function. Additionally, several dorsal and arthroscopic procedures use volar incisions that increase the risk of damaging the superficial radial nerve.^44^ Therefore, augmentation or imbrication of the joint capsule is often deemed as less technically challenging. Despite these concerns, the results of the various included techniques do not reflect the aforementioned arguments, indicating comparable positive outcomes for almost all the included surgical techniques. Apart from the included studies, various modifications to the Eaton and Littler's procedure or figure-of-eight tendon-looping techniques have been described for patients with minimal chondral damage or posttraumatic thumb CMC instability.^45^, ^46^, ^47^ Similarly, small case series with similar imbrication techniques have been reported in current literature, exhibiting favorable postoperative outcomes in line with the included studies.^27^^,^^28^^,^^48^

There are some limitations to this review. Despite pain and function being the most important parameters to consider after surgery, only eight of the included studies reported the VAS scores for pain. Additionally, pinch strength, grip strength, key pinch, and (Quick) DASH scores were variably reported across six, five, four, and three studies, respectively. However, the heterogeneity in the outcome measures used made the comparison of techniques difficult and prevented a meta-analysis from being performed. Additionally, this variability increased the difficulty in comparing the complication rates between studies owing to the differences in follow-up periods and varying definitions of the complications. Moreover, the discrepancy between the subjective and objective outcomes in the studies presented further complicated the analysis, as postoperative VAS pain scores did not consistently align with the elimination of postoperative pain or patient satisfaction.^7^^,^^35^^,^^42^ Notably, discrepancies in outcomes were observed even within the same technique. Moreover, the high patient satisfaction (90%) reported for the Eaton and Littler technique has been contradicted in the literature.^49^ Lastly, the evolution of nomenclature over the years has introduced challenges in determining and comparing different types of instability etiologies. The terms “pre-arthritic”, “early-arthritis,” or “instability” have diverse interpretations, resulting in different inclusion and exclusion criteria across studies. It can be challenging to discern whether CMC instability was the result of prior trauma, preexisting joint laxity, or both.

In conclusion, thumb CMC joint instability significantly impacts a patient's hand function and overall quality of life. The results of this review indicated that thumb CMC ligament reconstruction effectively alleviates pain and joint instability, leading to high patient satisfaction. Capsuloplasty, as a promising potential alternative to tendon-looping procedures, warrants further exploration. Future studies using standardized outcome measures and sufficient and homogenous sample sizes will provide evidence for patient counseling and also facilitate the identification of the optimal surgical technique for treating nontraumatic and nonarthritic thumb CMC instability.

## Declaration of competing interest

The authors declare there is no conflict of interests.

## Appendix Supplementary materials

Image, application 1
