# Supplementary material for: Ligament reconstruction in thumb carpometacarpal joint instability: A systematic review
Source: JPRAS Open. 2024 Jan 12;39:237–48. doi: 10.1016/j.jpra.2024.01.001 (PMC10843995; doi:10.1016/j.jpra.2024.01.001)
Supplement: Supplementary file 1 [file mmc1.docx]

**SUPPLEMENTARY FILE 1**

**Embase.com**

(('metacarpal bone'/de AND ('thumb'/de OR 'thumb injury'/exp)) OR (CMC-1 OR CMC1 OR CMCi OR CMC-I OR basal-joint* OR trapeziometacarpal* OR greater-multangular* OR ((metacarp* OR carpometacarp*) NEAR/6 (thumb* OR first* OR one OR 1))):ab,ti,kw) AND ('thumb reconstruction'/de OR 'joint instability'/de OR 'joint laxity'/de OR (instabili* OR reconstruct* OR repair* OR stabili* OR hypermobili* OR hyperflexib* OR laxi* OR hyperextensib* OR subluxat* OR dislocat* OR ligamentoplast*):ab,ti,kw) NOT [conference abstract]/lim AND [english]/lim

**Medline (ovid)**

((Metacarpal Bones/ AND Thumb/) OR (CMC-1 OR CMC1 OR CMCi OR CMC-I OR basal-joint* OR trapeziometacarpal* OR greater-multangular* OR ((metacarp* OR carpometacarp*) ADJ6 (thumb* OR first* OR one OR 1))).ab,ti,kf.) AND (Joint Instability/ OR Joint Dislocations/ OR (instabili* OR reconstruct* OR repair* OR stabili* OR hypermobili* OR hyperflexib* OR laxi* OR hyperextensib* OR subluxat* OR dislocat* OR ligamentoplast*).ab,ti,kf.) NOT (news OR congres* OR abstract* OR book* OR chapter* OR dissertation abstract*).pt. AND english.lg.

**Web of Science**

TS=(((CMC-1 OR CMC1 OR CMCi OR CMC-I OR basal-joint* OR trapeziometacarpal* OR greater-multangular* OR ((metacarp* OR carpometacarp*) NEAR/5 (thumb* OR first* OR one OR 1)))) AND ((instabili* OR reconstruct* OR repair* OR stabili* OR hypermobili* OR hyperflexib* OR laxi* OR hyperextensib* OR subluxat* OR dislocat* OR ligamentoplast*))) NOT DT=(Meeting Summary OR Meeting Abstract) AND LA=English

**Cochrane Central**

((CMC-1 OR CMC1 OR CMCi OR CMC-I OR basal-joint* OR trapeziometacarpal* OR greater-multangular* OR ((metacarp* OR carpometacarp*) NEAR/6 (thumb* OR first* OR one OR 1))):ab,ti,kw) AND ((instabili* OR reconstruct* OR repair* OR stabili* OR hypermobili* OR hyperflexib* OR laxi* OR hyperextensib* OR subluxat* OR dislocat* OR ligamentoplast*):ab,ti,kw) NOT "conference abstract":pt

**Google Scholar / Publish or Perish**

CMC1|CMCi|trapeziometacarpal|'basal joint'|'greater multangular'|'metacarpal|carpometacarpal|CMC thumb|one|1|i'|'first metacarpal|carpometacarpal' instability|reconstruction|repair|stability|hypermobility|hyperflexibility|laxity|hyperextensibility

**SUPPLEMENTARY FILE 3:** Quality Assessment

| Item | 1 | 2 | 3 | 4 | 5 | 6 | 7 | 8 | 9 | 10 | 11 | 12 | 13 | 14 | Rating |
| --- | --- | --- | --- | --- | --- | --- | --- | --- | --- | --- | --- | --- | --- | --- | --- |
| **Quality Assessment of Controlled Intervention Studies** | | | | | | | | | | | | | | |  |
| Spekreijse, 2016 (RCT) | Yes | Yes | Yes | No | No | Yes | Yes | Yes | Yes | Yes | Yes | No | Yes | Yes | Fair |
| **Quality Assessment Tool for Before-After (Pre-Post) Studies With No Control Group** | | | | | | | | | | | | | | |  |
| Eaton, 1984 | Yes | Yes | Yes | Yes | Yes | Yes | No | NR | No | No | No | NA |  |  | Poor |
| Lane, 2001 | Yes | Yes | Yes | NR | Yes | Yes | Yes | NR | NR | No | No | NA |  |  | Poor |
| Chu, 2009 | Yes | Yes | Yes | NR | No | Yes | Yes | NR | NR | Yes | No | NA |  |  | Poor |
| Spekreijse, 2016 (Cohort) | Yes | Yes | Yes | Yes | Yes | Yes | Yes | NR | Yes | Yes | No | NA |  |  | Fair |
| Kato, 2020 | Yes | Yes | Yes | NR | No | Yes | Yes | NR | Yes | No | No | NA |  |  | Poor |
| Kronlage, 2023 | Yes | Yes | Yes | Yes | Yes | Yes | Yes | NR | NA | Yes | No | NA |  |  | Fair |
| **Quality Assessment Tool for Observational Cohort and Cross-Sectional Studies** | | | | | | | | | | | | | | |  |
| Biddulph, 1985 | No | Yes | NR | Yes | No | Yes | Yes | NA | Yes | NA | No | NA | NR | No | Poor |
| Stauffer, 2020 | Yes | Yes | Yes | Yes | No | Yes | Yes | NA | Yes | NA | Yes | NA | NA | No | Fair |
| Pecache, 2022 | Yes | Yes | No | Yes | No | Yes | Yes | NA | Yes | NA | Yes | NA | NA | No | Poor |
| Koehler, 2021 | Yes | Yes | Yes | Yes | No | Yes | Yes | NA | Yes | NA | Yes | NA | NA | No | Fair |
| Freedman, 1999 | Yes | Yes | Yes | No | No | Yes | Yes | NA | Yes | NA | Yes | NA | NA | No | Poor |

*From:* National Heart, Blood and Lung Institute. Study quality assessment tools. <https://www.nhlbi.nih>.gov/health-topics/study-quality-assessment-tools. (June 14th).

**SUPPLEMENTARY FILE 4:** ICHAW Classification

| **Grade** | **Definition, to occur within the final time point of the relevant track** |
| --- | --- |
| Grade I: | Any deviation from the normal treatment course without the need for surgical, endoscopic and radiological interventions. Acceptable therapeutic regimens are: extra analgesics and additional hand therapy/ splinting/ cast. This grade incudes e.g.: tendinitis, scar tenderness, temporary sensory disturbances, etc.  Complex Regional Pain Syndrome is excluded from this grade (see Grade III-C). |
| Grade II: | Any deviation from the normal treatment course requiring antibiotics, steroid injections or other pharmacological treatment not listed in Grade I. Also included are wound infections and hematoma’s not needing anesthesia. Complex Regional Pain Syndrome is excluded from this grade (see Grade III-C). |
| Grade III:  A:  B:  C: | Any deviation from the normal treatment course requiring surgical, endoscopic or radiological intervention. Also, this includes tendinitis, scar tenderness, persistent pain, etc. not responding to conservative therapy, drugs or injections.  Minor surgical intervention under local anesthesia (e.g. irritating K wire, suture removal subcutaneously)  Major surgical intervention under regional or general anesthesia (e.g. repeat surgery, tenolysis, neurolysis, nerve repair or surgery for tendon rupture, breaking of plate, non-union, initial prosthesis failure)  Complex Regional Pain Syndrome, diagnosed using Budapest* criteria, independent of the initiated treatment |

*From***:** ICHOM. The International Consortium for Health Outcomes Measurement. Standard Set for Hand & Wrist Conditions. Accessed June 14th 2023. <https://www.ichom.org/portfolio/hand-and-wrist-conditions/>

**SUPPLEMENTARY FILE 5:** Classification of strength of evidence by Jovell and Narvarro-Rubio

| **Level** | **Strength of Evidence** | **Type of Study Design** |
| --- | --- | --- |
| I | Good | Meta-analysis of randomized controlled trials |
| II |  | Large-sample randomized controlled trials (N > 25 for each group) |
| III | Good to fair | Small-sample randomized controlled trials (N < 25 for each group) |
| IV |  | Non-randomized controlled prospective trials |
| V |  | Non-randomized controlled retrospective trials |
| VI | Fair | Cohort studies |
| VII |  | Case-control studies |
| VIII | Poor | Noncontrolled clinical series; descriptive studies |
| IX |  | Anecdotes or case reports |
